# Reduction of EEG Theta Power and Changes in Motor Activity in Rats Treated with Ceftriaxone

**DOI:** 10.1371/journal.pone.0034139

**Published:** 2012-03-30

**Authors:** Michele Bellesi, Vladyslav V. Vyazovskiy, Giulio Tononi, Chiara Cirelli, Fiorenzo Conti

**Affiliations:** 1 Department of Experimental and Clinical Medicine, Section of Neuroscience and Cell Biology, Università Politecnica delle Marche, Ancona, Italy; 2 Department of Psychiatry, University of Wisconsin at Madison, Madison, Wisconsin, United States of America; 3 Fondazione di Medicina Molecolare, Università Politecnica delle Marche, Ancona, Italy; Instituto de Neurociencias de Alicante UMH-CSIC, Spain

## Abstract

The glutamate transporter GLT-1 is responsible for the largest proportion of total glutamate transport. Recently, it has been demonstrated that ceftriaxone (CEF) robustly increases GLT-1 expression. In addition, physiological studies have shown that GLT-1 up-regulation strongly affects synaptic plasticity, and leads to an impairment of the prepulse inhibition, a simple form of information processing, thus suggesting that GLT-1 over-expression may lead to dysfunctions of large populations of neurons. To test this possibility, we assessed whether CEF affects cortical electrical activity by using chronic electroencephalographic (EEG) recordings in male WKY rats. Spectral analysis showed that 8 days of CEF treatment resulted in a delayed reduction in EEG theta power (7–9 Hz) in both frontal and parietal derivations. This decrease peaked at day 10, i.e., 2 days after the end of treatment, and disappeared by day 16. In addition, we found that the same CEF treatment increased motor activity, especially when EEG changes are more prominent. Taken together, these data indicate that GLT-1 up-regulation, by modulating glutamatergic transmission, impairs the activity of widespread neural circuits. In addition, the increased motor activity and prepulse inhibition alterations previously described suggest that neural circuits involved in sensorimotor control are particularly sensitive to GLT-1 up-regulation.

## Introduction

The amino acid L-glutamate (Glu) is the major excitatory neurotransmitter in the mammalian central nervous system, and is involved in most aspects of normal brain function, including fast excitatory signaling, synaptogenesis, and synaptic plasticity [1], [2]. Extracellular Glu levels are regulated by a group of Glu transporters (GluTs) that take up Glu from extracellular space, preventing its accumulation. Five GluTs have been characterized in the mammalian central nervous system: GLAST (EAAT1; SLC1A3), GLT-1 (EAAT2; SLC1A2), EAAC1 (EAAT3; SLC1A1), EAAT4 (SLC1A6) and EAAT5 (SLC1A7); of these, GLT-1 exhibits the highest level of expression, is responsible for the largest proportion of total Glu transport and its functional inactivation raises extracellular Glu to toxic levels [2]–[9]. GLT-1 is expressed by astrocytes [10]–[14], and, albeit at lower levels, by neurons [13]–[17]. In both astrocytic processes and axon terminals, most GLT-1a is perisynaptic, i.e. in the plasma membrane region extending 200–250 nm from the edge of the active zone [13], a position suitable for modulating Glu concentration in the cleft.

Due to its localization, GLT-1 controls the glutamatergic transmission by regulating the activation of the receptors mainly expressed at perisynaptic sites, thus playing an important role in synaptic physiology and pathophysiology [9], [18]. Several diseases indeed have been associated to changes of GLT-1 expression [1], [19]–[21], and more recent observations suggest that GLT-1 could be an ideal pharmacological target to prevent those conditions characterized by increased levels of extracellular Glu [22]–[24].

Rothstein and colleagues have recently shown that ceftriaxone (CEF) increases robustly and specifically GLT-1 expression and function [22]. Using this tool, we recently characterized GLT-1 up-regulation in different brain regions, and showed that CEF robustly increases GLT-1 expression in neocortex, hippocampus, striatum and thalamus. In addition, physiological studies have shown that GLT-1 up-regulation strongly affects the efficacy of the glutamatergic transmission [18], and leads to an impairment of the prepulse inhibition, a simple form of information processing [25], [26]. Altogether, these data suggest that CEF-induced GLT-1 over-expression has widespread effects on brain's functions involving large populations of neurons. To test this possibility, we assessed whether CEF treatment affects cortical activity by performing chronic electroencephalographic (EEG) recordings coupled with videorecordings in rats before and after CEF treatment.

## Results

### Ceftriaxone reduces theta (7–9 Hz) power

**Figure 1:**
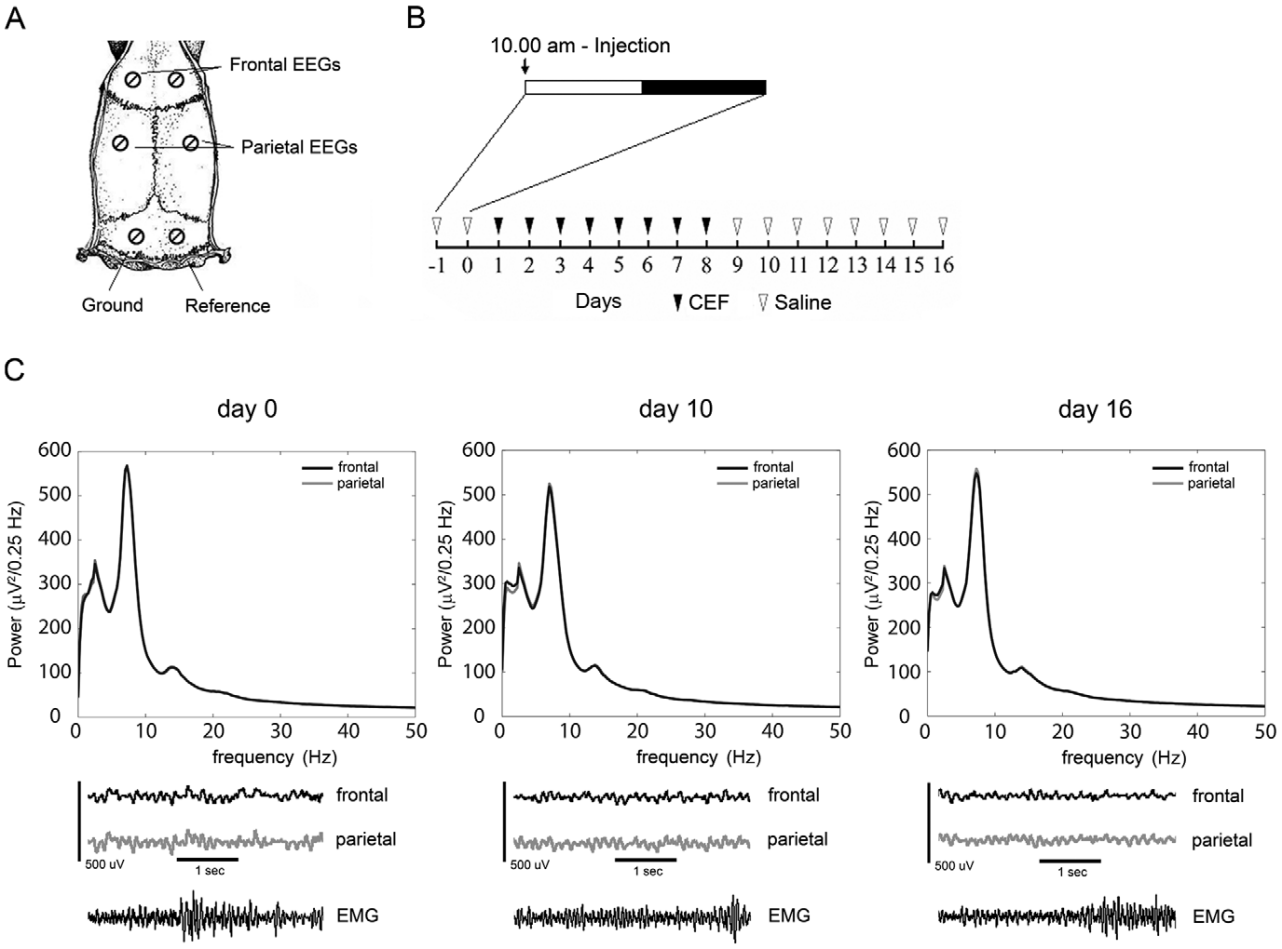
Lack of pathological elements after ceftriaxone treatment. **A.** Schematic description of electrodes location. **B.** Saline and CEF Treatment schedule. Day 0 represents the baseline. **C.** Waking absolute spectra, raw EEG and EMG signals of baseline, day 10 and day 16. Signals appeared stable across the entire length of the experiment and the signal quality was not affected by CEF treatment.

**Figure 2:**
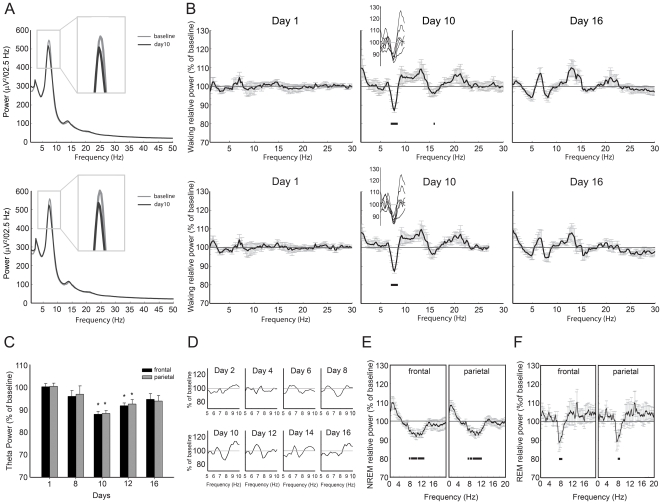
Power spectra analysis following ceftriaxone administration. **A.** Reduction in theta power at day 10. Waking mean absolute power spectra of Day 0 and Day 10 for frontal (above) and parietal (below) EEG channel. **B.** Power spectra analysis relative to the baseline illustrating a reduction of theta power at Day 10 (shown in detail for single animals in the small inset) and a return to the baseline eight days after CEF withdrawal (Day 16) in frontal (above) and parietal (below) EEG channels. Statistical significance (p<0.05) is represented by black dots. Values are mean ± sem. **C.** Time course analysis of relative spectra (7–9 Hz frequency band) showing a significant reduction of theta power for frontal and parietal channels two days (Day 10) and four days (Day 12) after CEF withdrawal compared to the baseline (Day 0). *p<0.05. Values are mean ± sem. **D.** Example of power spectrum relative to the baseline for a representative animal during the entire length of the experiment. **E.** Frontal and parietal relative spectra showing a broad band (7–13 Hz) reduction in power during NREM sleep at day 10. Statistical significance (p<0.05) is represented by black dots. Values are mean ± sem. **F.** Frontal and parietal relative spectra showing a reduction in theta power during REM sleep at day 10. Statistical significance (p<0.05) is represented by black dots. Values are mean ± sem.

Analysis of EEG traces did not show pathological elements (e.g., epileptic discharges or gross signal modifications) after CEF treatment (Figure 1). Power spectra analysis carried out on waking epochs at different time points showed that CEF administration was associated to a reduction (−11.4±1.2% frontal, −10.9±1.2% parietal) in theta power (7–9 Hz) (Figure 2A). The analysis was performed by dividing the EEG spectrum in 200 bins (1–200, frequency range 0.25–50 Hz, resolution 0.25 Hz) and comparing each bin across the different time points with a repeated-measure ANOVA. Statistically significant bins were further compared to the respective baseline value (day 0) by Dunnett's *post-hoc* test. The analysis showed that no significant differences were present at day 1, indicating that CEF did not affect EEG after a single injection. However, a significant cluster of bins corresponding to frequencies ranging between 7.5 Hz and 8.5 Hz was evident in both frontal and parietal channels at day 10 (p<0.05), i.e., two days after CEF withdrawal. Other frequency bands were not affected. At day 16, the same analysis did not show any significant difference, indicating that the effect of CEF on EEG was reversible (Figure 2B). Extending the analysis to different time points and taking into account a specific band (7–9 Hz), we showed that CEF treatment had a significant effect on EEG [F(5,25) = 4.6, p<0.01 frontal channel, F(5,25) = 3.8, p<0.05 parietal channel]. *Post-hoc* analysis of single time points compared to the baseline showed that theta power reduction started at day 8, even if not significantly, peaked at day 10, persisted for a few days (days 10 and 12 both p<0.05), and then faded at day 16 (Figure 2C and D). Although our study was mainly focused on waking, we also analyzed the EEG power spectra during sleep, and found significant differences for NREM and REM sleep at day 10. Power spectrum analysis, performed on frontal and parietal channels, showed a decrease in power for frequencies ranging between 7 and 13 Hz for NREM sleep, whereas a theta reduction, similar to the one demonstrated for waking, was documented for REM sleep (Figure 2E and F).

### Ceftriaxone treatment is associated to an increase of motor activity

**Figure 3:**
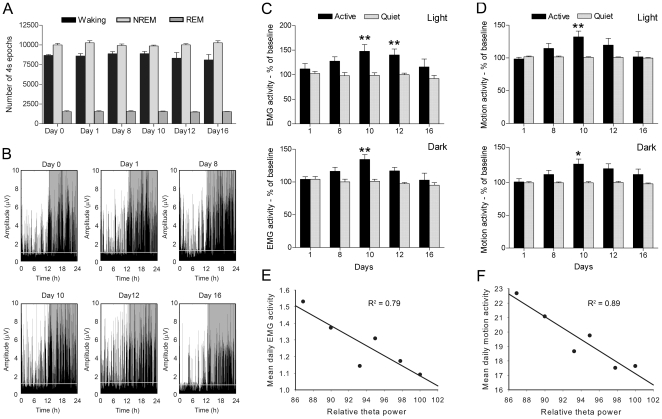
Effects of ceftriaxone treatment on motor activity. **A.** Time-course of the amount of time expressed in 4s epochs spent in waking, NREM and REM sleep. Values are expressed as mean ± sem. **B.** Example of EMG activity during the entire length of the experiments. Note the intense activity after the end of CEF treatment. The grey line represents the threshold above and below which the motor activity is identified as active waking or quiet waking, respectively. **C–D.** Quantitative analysis of EMG activity (C) and Motion activity (D) during active and quiet waking for light and dark periods. Values are relative to the baseline (day 0) and expressed as mean ± sem. * (p<0.05), ** (p<0.01). **E–F.** Negative correlation between the time-course of relative theta power and the EMG (E) or Motion activity (F).

Since changes in theta activity are strongly associated to changes in motor behavior in rats [27]–[29], we investigated whether our animals showed motor abnormalities due to CEF treatment. Firstly, we determined whether CEF treatment modifies the time spent in waking and sleep, in order to rule out that changes in motor activity were simply a consequence of changes in wake duration. To this aim, we performed a quantitative analysis of the total amount of waking, NREM and REM sleep epochs scored, and found no significant differences, neither during the 10 days of treatment nor afterwards [waking: F(5,25) = 0.98, ns; NREM: F(5,25) = 1.2, ns; REM: F(5,25) = 0.26, ns; Figure 3A].

Since the amount of waking and sleep time did not change with CEF treatment, we next evaluated whether the type or “intensity” of motor activity have been affected by CEF treatment. We therefore analyzed both EMG traces and video recordings before and after treatment, and quantified EMG and Motion activity, respectively. By setting a threshold equal to 95^th^ percentile of EMG or Motion activity during all NREM sleep episodes (see Methods), we assessed the amount of motor activity occurring during active and quiet waking. For active waking, time-course analysis of EMG activity during the light period showed a significant effect of time [F(5,25) = 5.8; p<0.01], and *post-hoc* comparisons showed a significant increase on day 10 and 12 compared to baseline (p<0.01 for each time points). In addition, analysis of EMG activity during the dark period showed a similar significant effect of time [F(5,25) = 7.4; p<0.001] with a significant increase on day 10 compared to baseline (p<0.01) at *post-hoc* comparisons. For quiet waking, the same analysis carried out for both dark and light period resulted not significant [light: F(5,25) = 1.08, ns; dark F(5,25) = 1.04, ns] (Figure 3C). EMG data were confirmed by video recordings analysis. For active waking, motion activity measured on the same animals was increased after the end of treatment for both light and dark periods. Time-course analysis showed an overall effect of time [F(5,25) = 4.1 p<0.01 for light period; F(5,25) = 3.93 p<0.01 for dark period] and subsequent comparisons confirmed a significant increase of activity at day 10 (p<0.01 for dark period, p<0.05 for light period) compared to the baseline. On the contrary, motion activity measured for quiet waking did not change through the course of the experiment [dark: F(5,25) = 0.93, ns; light: F(5,25) = 1.01, ns] (Figure 3D). Taken together, these data show that CEF treatment is associated to a time-limited increase of motor activity after treatment withdrawal with a peak at day 10. Next, we verified whether the time course of the increase of motor activity and theta power reduction were correlated. We found that both EMG and Motion activity were negatively correlated to theta power decrease (r = −0.89, p<0.05; r = −0.94, p<0.01, respectively, Figure 3E and F).

## Discussion

The main result reported here is that one week CEF treatment resulted in a delayed reduction in EEG theta power (7–9 Hz) in both frontal and parietal derivations. This decrease peaked at day 10, 2 days after the end of treatment, and disappeared by day 16. In addition, we found that the same CEF treatment increased motor activity, especially during those experimental days in which the EEG changes are more prominent.

Since EEG is sensitive to different environmental conditions and animal manipulations [30], we assessed the effects of CEF by using a within-subjects design, comparing data before and after CEF treatment. To rule out the possibility that EEG changes reflected the chronic effect of i.p. injections and/or treatment withdrawal rather than the actual effect of CEF, we continued to treat the animals with daily saline i.p. injections until the end of the experiment. In this way, the animals were exposed every day to the same manipulation for the entire length of the experiment, minimizing possible interferences of animal handling on the EEG analysis.

Although it has been well documented that CEF treatment can affect the expression of the major Glu transporter GLT-1 in rodents [22], [25], [31], [32], we did not measure GLT-1 levels in treated animals because of our experimental design. Nonetheless, using the same treatment schedule, we previously showed that CEF-induced GLT-1 modifications persist for at least four days after the end of treatment, and return to baseline level eight days after the end of treatment [25]. The delayed emergence of electrophysiological and behavioral changes relative to GLT-1 up-regulation, which can be documented at day 8 [25], may indicate that all the synaptic modifications observed and their functional consequences [18], [26] require time to affect the dynamics of large neuronal populations and lead to behavioral changes.

In rodents, theta oscillations are easily observed in the hippocampus, but can also be detected in other cortical and subcortical brain structures [33]–[35]. Historically, they have been associated to learning and memory and voluntary movements [36]–[40]. Lesions at different level of the hippocampal formation correlate to sizeable reductions of the hippocampal theta rhythm and to impairments of memory tasks [38], [41]–[43]. Parallel augmentation of long-term synaptic potentiation and theta activity was described by Maren and collaborators in a contextual fear-conditioning task [44], and more recent electrophysiological studies showed an increase of theta rhythm associated with induction of long-term potentiation [45], thereby suggesting a close relationship between theta oscillations and long-term synaptic plasticity [46]. Recently, Omrani and colleagues demonstrated that CEF-induced GLT-1 up-regulation at CA3 synapses impairs long-term plasticity, by preventing perisynaptic metabotropic receptors activation [18]. It is worth noting that our results are in line with these findings, and it is therefore conceivable to hypothesize that changes in synaptic plasticity can interfere with the normal function of the hippocampus, which in turn can generate a lower degree of synchronization, visible as a reduction in EEG theta power. Omrani and colleagues also showed that baseline fEPSPs were not affected by CEF treatment, indicating that the glutamatergic function is altered by GLT-1 up-regulation only when synapses undergo long-term plasticity [18]. It is therefore conceivable that AMPA receptors activation at the glutamatergic synapses, even in a condition of reduced synaptic plasticity, could ensure a normal EEG signal. This might explain why the observed decrease in theta activity was not associated with an overall decrease in EEG power over the entire range of frequencies.

It is worth noting that we also found modifications of NREM and REM sleep power spectrum at day 10. Specifically, the analysis of NREM power spectrum showed a significant reduction of a broad band ranging between 7 and 13 Hz, which includes the spindle frequency band, suggesting that other pattern generators beside the hippocampus might be affected by CEF treatment. Interestingly, in a previous study we found that the GLT-1 is up-regulated by CEF in several brain regions, including thalamus and cerebral cortex, brain regions crucial in spindles formation and propagation [47]. REM sleep normally is characterized by a robust theta activity in rodents, and several lines of evidence indicate that modifications in waking theta activity can be reflected in REM theta activity [48]–[50]. Along this line, we found a decrease in theta power during REM sleep resembling the one observed during waking, although less prominent. The combined theta reduction in waking and in REM sleep induced by CEF could be ascribable to an impairment of a common circuit promoting theta formation in the two behavioral states.

Rats treated with CEF showed an increase of motor activity occurring after the end of treatment, i.e., when EEG alterations were clearly evident. Reportedly, theta is the electrical sign of activity in a forebrain mechanism that is organizing higher voluntary motor acts [27], [51], [52]. Evidence suggests that there are two distinct types of hippocampal theta rhythm in behaving animals: the first (4–7 Hz) appears when animals are immobile or during repetitive acts such as sniffing or whiskers movements [53], [54]; the second one is more directly linked to voluntary motor behaviors (i.e., walking, running, rearing etc) and it is characterized by higher frequency (7–10 Hz) [29]. In physiological conditions, motion speed is associated to the frequency of theta rhythm, as indicated by studies reporting that the higher is the speed, the faster is frequency [28], [55], [56]. Moreover, electrophysiological studies demonstrated that theta activity recorded while the animal was running is larger than during walking, thus suggesting that the amplitude of theta rhythm increases as the intensity of movement increases [51]. On the contrary, our results show a reduction of EEG theta power when motor activity is increased. By assessing separately motor activity during active and quiet waking we found that only active waking (mostly characterized by exploratory behavior) was affected by CEF treatment, thereby revealing a dissociation between theta power and motor activity. Of note ketamine, another drug that affects glutamatergic transmission, when administered to mimic pathological states (i.e., schizophrenia) causes both an increase in motor activity and a decrease in EEG theta power [57]–[59]. Thus, it appears that the reduction of theta power, at least in non-physiological, drug-induced conditions, can be associated with an increase in motor activity. In addition, drug-induced motor activity is often mediated by aberrant activation of basal ganglia and cerebral cortex [60]. It is therefore possible that glutamatergic alterations also affecting those structures [25], [26] may contribute to the increase of motor activity we observed. In support to the possible co-occurrence of theta activity reduction and increased motor activity, we also asked whether the time courses of those alterations were correlated. We found indeed a significant correlation between the EEG changes and the motor modifications, supporting a link between them, although a causal relationship still remains to be established.

In addition, there is evidence that theta oscillations play a role in integrating sensorimotor information [52], [61]. It is well known that a simple measure to evaluate sensorimotor integration in animals and in humans is prepulse inhibition of the startle reflex [62], [63], which reflects the ability of the brain to temporarily adapt to a strong sensory stimulus when a preceding weaker signal is given to warn the organism [62], [63]. PPI is a neurological phenomenon regulated by a complex interplay between several brain structures, including cortical and subcortical sites [64]. Interestingly, we recently demonstrated that the PPI was impaired in rats treated with CEF and that this deficit was blocked by dihydrokainate, a selective GLT-1 inhibitor [25], thus suggesting that sensorimotor integration is affected by GLT-1 up-regulation.

Taken together, these data indicate that GLT-1 up-regulation, by modifying the efficacy of the glutamatergic transmission, impairs the activity of widespread neural circuits and that the reduction in EEG theta power could be its electrophysiological signature. In addition, the increased motor activity and PPI alterations previously described suggest that neural circuits involved in sensorimotor control are particularly sensitive to changes in the efficacy of glutamatergic transmission induced by GLT-1 up-regulation.

## Materials and Methods

### Ethics statement

Animal protocols followed the National Institutes of Health Guide for the Care and Use of Laboratory Animals, in accordance with institutional guidelines. They were reviewed and approved by the IACUC of the University of Wisconsin-Madison, and were inspected and accredited by AAALAC (Protocol M2006).

### Animals, surgery and treatment

Male WKY rats (n = 6, Harlan, 11–12 weeks old at time of surgery) were used. Under deep isoflurane anesthesia (1.5–2% volume), rats were implanted bilaterally with epidural screw electrodes over the frontal (B: +2 mm, L: 2 mm) and parietal cortex (B: −2 mm, L: 4 mm) and cerebellum (reference electrode and ground) for chronic EEG recordings. Electrodes were fixed to the skull with dental cement. Two stainless steel wires (diameter 0.4 mm) were inserted into neck muscles to record electromyogram (EMG) (Figure 1A).

After surgery, all rats were housed individually in transparent plexiglas cages (36.5×25×46 cm), and kept in sound-proof recording boxes for the duration of the experiment. Light and temperature were kept constant (LD 12∶12, light on at 10 am, 23±1°C; food and water were available *ad libitum* and replaced daily at 10 am). About seven days were allowed for recovery after surgery, and experiments were started only after animals were fully recovered. Animal protocols followed the National Institutes of Health Guide for the Care and Use of Laboratory Animals and were in accordance with institutional guidelines.

Rats were connected by a flexible cable to a commutator (Airflyte, Bayonne, NJ) and recorded continuously for 4 weeks using the Multichannel Neurophysiology Recording and Stimulation System (Tucker-Davis Technologies Inc., TDT).

Rats were treated intraperitoneally (i.p.) for two days with saline (day 1 was for habituation, day 2 was the baseline), followed by CEF (dissolved in saline [∼0.2 ml] and administered i.p. at a dose of 200 mg/kg; [25]) for 8 days, and then with saline i.p. for 8 days. All injections were administered at 10 am (Figure 1B).

### Data acquisition and analysis

#### EEG and EMG

EEG and EMG signals were amplified and filtered as follows: EEG: high-pass filter at 0.1 Hz; low-pass filter at 100 Hz; EMG: high-pass filter at 10 Hz; low-pass filter at 100 Hz. All signals were sampled and stored at 256 Hz resolution. EEG power spectra were computed by a Fast Fourier Transform routine for 4-s epochs (0.25 Hz resolution). For staging, signals were loaded with custom-made Matlab programs using standard TDT routines, and subsequently transformed into the EDF (European Data Format) with Neurotraces software. Waking, NREM sleep, and REM sleep were manually scored off-line (SleepSign, Kissei COMTEC, Matsumoto, Japan) in 4-s epochs according to standard criteria. Epochs containing artifacts, predominantly during active waking, were excluded from spectral analysis. Vigilance state could always be determined. EEG channels presenting artifacts or abnormal reduction of signal amplitude during the entire duration of the experiment were not included in the analysis. At the end, all animals had at least one valid channel from frontal and parietal cortices. Absolute spectra revealed a normal pattern in all the animals recorded before and after CEF treatment (Figure 1C).

EMG signals were loaded with custom-made Matlab programs and rectified amplitudes were calculated over 4-s epochs and used to quantify the activity of the animals. The magnitude of this index corresponded to the amount of motor activity that occurred during that 4-sec epoch. A threshold, below and above which all waking epochs were classified as quiet waking and active waking, respectively, was determined by calculating the 95^th^ percentile of EMG activity during all NREM sleep episodes [65]. This method accurately identified periods of relative inactivity during waking, in which the animals were quiet or performed little movements (such as head movements or postural adjustments), but staying at the same place, and more active periods in which animals were engaged in explorative activities.

#### Video-Recordings

Video recordings were performed continuously with infrared cameras (OptiView Technologies, Inc. Potomac Falls, VA) and stored in real time (AVerMedia Technologies, Inc. Milpitas, CA). A custom-made Matlab script was used for analysis. The program detected animal motion every second within a previously set monitored area (corresponding to the cage area), by calculating the numbers of pixels whose intensity changed over time. Specifically, it compared the last image with the following one and defined a value in percent of changes occurring every second. These values and the relative time were then daily saved in a txt report file and subsequently loaded with custom-made Matlab programs. Percentage values (Motion activity) for light and dark periods were processed separately, because cameras showed a different sensibility in detecting motion in different light conditions.

A threshold corresponding to the mean amount of pixels changing during NREM sleep was set to classify active versus quiet waking as for EMG studies.

#### Statistical analysis

For EEG analysis, mean absolute EEG spectrum values for each state were normalized by dividing each 0.25 Hz bin value by the total mean value. For time-course studies, EEG, EMG and Motion activity, all values at different time points during the experimental days were compared to the corresponding baseline (Day 0) values. Comparisons were performed by using a repeated-measure ANOVA, with time as a within factor, follow by Dunnett's post-hoc test or a paired t-test in presence of multiple or single comparisons, respectively. Pearson's correlation coefficient was calculated for correlative studies between EMG or motion activity and theta relative power. Alpha was set at 0.05.
